# Accessory instrumentation in flexible ureteroscopy: Evidence-based recommendation

**DOI:** 10.4103/0970-1591.39548

**Published:** 2008

**Authors:** Timothy Holden, Renato Nardi Pedro, Monoj Monga

**Affiliations:** Department of Urologic Surgery, University of Minnesota, Minneapolis, MN 55455, USA

**Keywords:** Endourology, flexible ureterocopy, ureteral stone

## Abstract

Instrumentation is the key to success in endourology. Indeed, endourology could be redefined as “enginurology” as the marriage between engineering and urology to develop instrumentation to improve patient outcomes is the key facilitator in the advancement of minimally invasive techniques. This review article will identify the evidence-base that supports our current recommendations for equipment used during ureteroscopy.

## INTRODUCTION

The advent and technological progression of flexible ureteropyeloscopy has led to more effective diagnostic and therapeutic interventions in endourology. Specifically, the innovations in fiberoptic technology, ureteroscope design, surgical technique, and accessory instrumentation has allowed for minimally invasive options for the treatment of upper tract urinary stones. Associated with this technological evolution is an ever-widening array of instruments available to endourologists. This review outlines the instrumentation we prefer, with corresponding evidence.

## INSTRUMENTATION

### Guide wire

The placement of a safety wire facilitates and maintains access to the upper urinary tract. Various guide wires have been developed with an array of physical parameters, such as length, diameter, tip flexibility, shaft rigidity, and surface coating. An ideal guide wire requires little force to flex in response to resistance encountered along a tortuous path, while contrarily requiring a large force to perforate through tissue. These properties were examined *in vitro* with nine commercially available guide wires by measuring the tip and shaft bending forces, pull force, and tip puncture force.[1] From these measured parameters, a “margin of error” can be extrapolated, defined as the difference between the ureter perforating force and the force required to bend the tip of the guide wire. The study demonstrated that the lubricous, soft-tip nitinol Glidewire (Boston Scientific Corp., Natick, MA, USA) is the safest wire for initial access to the ureter, as it is less likely to perforate and more likely to bend when a point of obstruction is encountered. In contrast, the super-stiff guide wire is the least likely to slip out inadvertently. Therefore, we prefer the Sensor wire (Boston Scientific), which is a hybrid wire that combines these different features. It contains three segments: a smooth, hydrophilic distal tip for bypassing impacted ureteral stones, a kink-resistant body (nitinol core with polytetrafluoroethylene coating), and a flexible proximal tip for back-loading of the wire through the working channel of the ureteroscope.

### Ureteral access sheath

Ureteral access sheaths are routinely placed over a super-stiff working guide wire to facilitate the insertion of the ureteroscope into the upper urinary tract during multiple stone fragment extractions. The use of a ureteral access sheath has been demonstrated to help facilitate ureteral re-entry, decrease operative time and cost, minimize patient morbidity, and optimize overall success with intrarenal ureteroscopic surgery.[2] The access sheath also allows efflux of irrigant fluid through the sheath and around the ureteroscope, maintaining intrapelvic pressures below 20 cm water with pressurized irrigant fluid up to 200 cm water.[3] In a randomized comparison of two ureteral access sheaths, the Cook Flexor sheath (Cook Urological, Bloomington, IN, USA) was rated superior to the Applied Access Forte XE (Applied Medical, Rancho Santa Margarita, CA, USA) with regards to ease of placement, instrument passage, and stone extraction.[4] The Cook Flexor sheath is also more resistant to both buckling at the ureteral orifice and kinking after removal of the inner dilator.[5] *In vitro* studies have further demonstrated that the Cook Flexor sheath has one of the largest inner diameters in the most common bending positions of straight and 30° bends, which further facilitates stone extraction.[6]

### Ureteral balloon dilator

Ureteral balloon dilation is commonly used to overcome ureteral strictures, but is also utilized in <5% of cases when the ureteral access sheath will not advance to the site of pathology. An ideal ureteral balloon would dilate to 100% of the expected diameter while under any amount of radial constrictive force. A comparative *in vitro* study compared the dynamics of ureteral balloon expansion under varying, extrinsic compressive forces, and inflation pressures.[7] At inflation burst pressure, the Cook Ascend AQ (Cook Urological) was the most reliable balloon, achieving and maintaining over 100% of the expected inflation diameter with minimal pressure and a small coefficient of variation.

### Flexible ureteroscope

Upon placement of a guide wire and ureteral access sheath, an appropriate flexible ureteroscope is selected for insertion into the collecting system. A comparison of commercially available flexible ureteroscopes concluded that the unique fused quartz bundle of the Wolf ureteroscopes provides better optics than glass fiberoptic bundles.[8] The Wolf Viper (Richard Wolf Endoscopy, Vernon Hills, IL, USA) 7.56Fr was shown to have twofold greater resolution than the other flexible ureteroscopes, as defined by the imaging system's ability to distinguish object detail. In addition, in vitro evaluations of scope manipulation have demonstrated that the Wolf Viper is superior at accessing all calycles in a hydronephrotic kidney model.[9]

### Intracorporeal lithotrite

Upon visualization of the stone with the flexible ureteroscope, intracorporeal lithotripsy is used for large stone fragmentation. Holmium laser lithotripsy has been shown to fragment all compositions of urinary calculi, and to produce smaller stone fragments than pneumatic or electrohydraulic lithotripsy.[10] In a comparison study, the stone-free rates both at the end of the ureteroscopy and 3-months postprocedure were significantly higher for holmium *vs*. electrohydraulic lithotripsy.[11] Holmium laser also results in a lower incidence of both secondary procedures and stone migration for upper ureteric calculi as compared to pneumatic lithotripsy.[12] Performance and safety studies of commercially available holmium laser fibers demonstrated that the Dornier Lightguide 200 was the most likely of small fibers (200-273 mm) to fracture and damage a flexible ureteroscope, while the Lumenis 272 (Coherent) and the Innova Quartz 400 (Gyrus-ACMI) were the most durable in their size class.[13]

### Stone retrieval devices

A variety of stone retrieval devices are utilized in ureteroscopy under different circumstances. Important properties of stone retrieval baskets include basket visibility during stone manipulation, sufficient radial force to open in the ureter, and the ability to capture, retain, or (if necessary) disengage a stone. In general, *in vitro* studies have shown that the basket configuration and linear opening dynamics of the Cook NCircle 2.2F (Cook Urological) best facilitate efficient stone capture from ureteral and calyceal models.[14–16] The Cook NCircle was shown to attain the most rapid target-basket-width compared to 12 other baskets, suggesting a more controlled view when opening.[17] Specific variables, however, can influence which stone retrieval device is most appropriate for a specific procedure. The Microvasive Zerotip 1.9F (Boston Scientific) has a larger basket dimension that best facilitates the release of a stone after capture, and is used if a stone must be repositioned from the lower pole to an upper pole calyx.[14] The Cook NCompass (Cook Urological) has a webbed configuration that best facilitates the capture of stones as small as 1 mm in size, and is used when multiple small stone fragments are present. The Stone Cone (Boston Scientific) consists of concentric coils which, when placed proximal to calculi, act to prevent proximal retropulsion of stone fragments during lithotripsy.[18] The device has been shown clinically to reduce the incidence of residual stone fragments of over 3 mm in size.[19] The Cook NCircle 2.2F (Cook Urological) basket wires are more pliable, and can be distorted with gentle pressure until a stone fragment adherent to the renal papilla is surrounded and grasped. Gentle partial opening and closing of the basket can assist in stone disengagement, as well as forceful irrigation to wash a stone that is difficult to access into the basket. The 1.5F Sacred Heart Halo (Sacred Heart Medical, MN, Minnetonka, USA) allows rotation of an engaged stone via a rotary wheel on the basket handle, and is utilized if a stone may be too large for removal down the ureter. Furthermore, for entrapped stones, a 200-μm laser fiber can be passed alongside the Halo basket, and simultaneous laser lithotripsy/stone rotation can be performed for more complete stone fragmentation.[20] It is important to emphasize that stone capture with subsequent laser lithotripsy should be considered the exception, to be used only in the situation of an entrapped stone. Otherwise, laser lithotripsy prior to stone capture is advocated as a safer approach.

### Ureteral stent

Following the procedure, the ureter is inspected with the flexible ureteroscope as the ureteral access sheath is removed. Ureteral stents are used for both the prevention and treatment of ureteral obstruction following ureteroscopy. A ureteral stent is always left after placement of a ureteral access sheath, as anecdotal experience with not stenting in this situation is a higher prevalence of significant transient pain for 24 h. The Bard Inlay 6F ureteral stent (Bard Medical) has been associated with less severe urinary symptoms than other ureteral stents.[21]

## CONCLUSION

The growing prevalence of flexible ureteropyeloscopy as a diagnostic and therapeutic tool for endourologists is due in large part to the dramatic evolution in instrument design and technology. With this evolution comes an ever-widening array of tools and technology available to the endourologists. Having the right instrument in the right situation will help facilitate positive operative outcomes.

## References

[1] Clayman M, Uribe CA, Eichel L, Gordon Z, McDougall EM, Clayman RV (2004). Comparison of guide wires in urology. Which, when and why?. J Urol.

[2] Kourambas J, Byme RR, Preminger GM (2001). Does a ureteral access sheath facilitate ureteroscopy?. J Urol.

[3] Rehman J, Monga M, Landman J, Lee DI, Felfela T, Conradie MC, Srinivas R, Sundaram CP, Clayman RV (2003). Characterization of intrapelvic pressure during ureteropyeloscopy with ureteral access sheaths. Urology.

[4] Monga M, Best S, Venkatesh R, Ames C, Lieber D, Vanlangendock R, Landman J (2004). Prospective randomized comparison of 2 ureteral access sheaths during flexible retrograde ureteroscopy. J Urol.

[5] Monga M, Gawlik A, Durfee W (2004). Systematic evaluation of ureteral access sheaths. Urology.

[6] Beiko DT, Watterson JD (2005). From bench to ureteroscopy: a comparison of ureteral access sheath physical properties. J Endourol.

[7] Hendlin K, Lund B, Dockendorf K, Ramani R, Monga M (2005). Radial dilation of ureteral balloons: A comparative *in vitro* analysis. J Endourol.

[8] Abdelshehid C, Ahlering M, Chou D, Park HK, Basillote J, Lee D, Kim I, Eichel L, Protsenko D, Wong B, McDougall E, Clayman RV (2005). Comparison of flexible ureteroscopes: deflection, irrigant flow and optical characteristics. J Urol.

[9] Monga M, Weiland D, Pedro RN, Lynch AC, Anderson K (2007). Intrarenal manipulation of flexible ureteroscopes: A comparative study. BJU Int.

[10] Teichman JM, Vassar GJ, Bishoff JT, Bellman GC (1998). Holmium:YAG lithotripsy yields smaller fragments than lithoclast, pulsed dye laser or electrohydraulic lithotripsy. J Urol.

[11] Teichman JM, Rao RD, Rogenes VJ, Harris JM (1997). Ureteroscopic management of ureteral calculi: Electrohyraulic versus holmium: YAG lithotripsy. J Urol.

[12] Maheshwari PN, Trivedi N, Kaushik S (2003). Ureteroscopic management of upper ureteric calculi: Holmium lithotripsy vs. pneumatic lithotripsy (abstract). J Endourol.

[13] Knudsen BE, Glickman RD, Stallman KJ, Maswadi S, Chew BH, Beiko DT, Denstedt JD, Teichman JM (2005). Performance and safety of holmium: YAG laser optical fibers. J Endourol.

[14] Monga M, Hendlin K, Lee C, Anderson JK (2004). Systematic evaluation of stone basket dimensions. Urology.

[15] Lukasewycz S, Skenazy J, Hoffman N, Kuskowski M, Hendlin K, Monga M (2004). Comparison of nitinol tipless stone baskets in an *in vitro* caliceal model. J Urol.

[16] Lukasewycz S, Hoffman N, Botnaru A, Deka PM, Monga M (2004). Comparison of tipless and helical baskets in an *in vitro* ureteral model. Urology.

[17] Hendlin K, Lee C, Anderson JK, Monga M (2004). Radial dilation force of tipless and helical stone baskets. J Endourol.

[18] Dretler SP (2001). The stone cone: A new generation of basketry. J Urol.

[19] Desai MR, Patel SB, Desai MM, Kukreja R, Sabnis RB, Desai RM, Patel SH (2002). The Dretler stone cone: A device to prevent ureteral stone migration - The initial clinical experience. J Urol.

[20] Canales BK, Ramani A, Monga M (2006). A new spin on the entrapped ureteral calculus. J Endourol.

[21] Lee C, Kuskowski M, Premoli J, Skemp N, Monga M (2005). Randomized Evaluation of ureteral stents using validated symptom questionnaire. J Endourol.

